# Supplementary material for: Effect of Size and Loading of Retinoic Acid in Polyvinyl Butyrate Nanoparticles on Amelioration of Colitis
Source: Polymers (Basel). 2021 May 2;13(9):1472. doi: 10.3390/polym13091472 (PMC8124360; doi:10.3390/polym13091472)
Supplement: Supplementary file 1 [file polymers-13-01472-s001.zip › polymers-1193079-supplementary.pdf]

Supporting Information

Effect of size and loading of retinoic acid in polyvinyl butyrate nanoparticles on amelioration of colitis

Jinting Li [1,†], Yunmei Mu [1,†], Yiwei Liu [2], Akihiro Kishimura [1,2,3,4], Takeshi Mori [1,2,3*], Yoshiki Katayama [1,2,3,4,5*]

[1] Graduate School of Systems Life Sciences, Kyushu University, 744 Motooka, Nishi-ku, Fukuoka 819-0395, Japan
[2] Department of Applied Chemistry, Faculty of Engineering, Kyushu University, 744 Motooka, Nishi-ku, Fukuoka 819-0395, Japan
[3] Center for Future Chemistry, Kyushu University, 744 Motooka, Nishi-ku, Fukuoka 819-0395, Japan
[4] International Research Center for Molecular System, Kyushu University, 744 Motooka, Nishi-ku, Fukuoka 819-0395, Japan
[5] Department of Biomedical Engineering, Chung Yuan Christian University, 200 Chung Pei Rd., Chung Li, 32023, Taiwan
* Correspondence:
Takeshi Mori − Graduate School of Systems Life Sciences, Department of Applied Chemistry, Faculty of Engineering, and Center for Future Chemistry, Kyushu University, Fukuoka 819-0395, Japan; Email: mori.takeshi.880@m.kyushu-u.ac.jp; Tel.: 092-802-2850
Yoshiki Katayama − Graduate School of Systems Life Sciences, Department of Applied Chemistry, Faculty of Engineering, Center for Future Chemistry, and International Research Center for Molecular System, Kyushu University, Fukuoka 819-0395, Japan; Department of Biomedical Engineering, Chung Yuan Christian University, Taoyuan 32023, Taiwan; Email: ykatatcm@ mail.cstm.kyushu-u.ac.jp; Tel.: 092-802-2850
† These authors equally contributed to this work.

Table S1. PVBu NPs characteristics in cell culture medium. Data are expressed as mean ± S.D (n=3).

PVBu NPs name	cell culture medium with serum		cell culture medium without serum	
	Size (nm)	PDI	Size (nm)	PDI
NP1	124 ± 0.9	0.20 ± 0.01	123 ± 1.7	0.21 ± 0.02
NP2	208 ± 0.8	0.27 ± 0.03	207 ± 0.7	0.27 ± 0.01
NP2-ATRA	200 ± 0.2	0.17 ± 0.04	205 ± 1.2	0.21 ± 0.01
NP1-DIO	125 ± 1.0	0.18 ± 0.01	124 ± 0.7	0.19 ± 0.02
NP2-DIO	214 ± 0.9	0.24 ± 0.01	211 ± 1.8	0.28 ± 0.03
